# Identification of Xq22.1-23 as a region linked with hereditary recurrent spontaneous abortion in a family

**Published:** 2013-08

**Authors:** Sahar Shekouhi, Fatemeh Baghbani, Mohammad Hasanzadeh Nazar-Abadi, Tayebeh Hamzehloie, Mohammad Reza Abbaszadegan, Nafiseh Saghafi, Reza Raoofian, Javad Zavar Reza, Shahab Ahmadzadeh, Mohammad Amin Tabatabaiefar, Majid Mojarrad

**Affiliations:** 1 *Department of Medical Genetics, School of Medicine, Mashhad University of Medical Sciences, Mashhad, Iran.*; 2 *Department of Gynecology, Qaem Hospital, Mashhad University of Medical Sciences, Mashhad, Iran.*; 3 *Department of Medical Genetics, School of Medicine, Ahvaz Jundishapur University of Medical Sciences, Ahvaz, Iran.*; 4 *Department of Biochemistry, School of Medicine, Shahid Sadoughi University of Medical Sciences, Yazd, Iran.*

**Keywords:** *X-linked*, *Recurrentspontaneousabortion*, *Linkage*

## Abstract

**Background:** Recurrent spontaneous abortion (RSA) is one of the most common health complications with a strong genetic component. Several genetic disorders were identified as etiological factors of hereditary X linked RSA. However, more genetic factors remain to be identified.

**Objective:** In this study we performed linkage analysis on a large X linked RSA pedigree to find a novel susceptibility locus for RSA.

**Materials and Methods:** A linkage scan using 11 microsatellites was performed in 27 members of a large pedigree of hereditary X-linked RSA. Two point parametric Linkage was performed using Superlink v 1.6 program.

**Results:** Evidence of linkage was observed to markers at Xq23, DXS7133 and at Xq22.1 DXS101, with LOD score of 3.12 and 1.60, respectively.

**Conclusion:** Identified locus in this study may carry a responsible gene in RSA. Narrowing down of this region may leads to identification of this gene.

This article extracted from M.Sc. thesis. (Sahar Shekouhi)

## Introduction

Spontaneous abortion is the fate of approximately 15% of all clinically recognized pregnancies in the general population (1-5). This disorder is a sporadic phenomenon, and usually does not repeat in a family, however, 1-2% of couples who try to conceive suffer from recurrent spontaneous abortion (6). 

Recurrent spontaneous abortion (RSA) is defined as three or more consecutive pregnancy losses prior to 20-22 week of gestation (7). RSA is a heterogeneous disease, and several factors have been identified as the etiologies of this disease. Each of these factors causes a small proportion of the total cases including hormonal disorders (20%), immunological abnormalities (20%), gynecologic anatomical abnormalities (15%), genetic diseases (2-5%), and infections (1-2%) (5, 8, 9). The specific cause of RSA remains unknown in almost 50% of affected couples (10-12). Unknown single-gene or polygenic disorders, involved in fundamental cellular and reproductive processes, can cause idiopathic recurrent euploid losses.

RSA patients often occur as isolated case in families; however almost rarely occur as a hereditary complication in pedigrees. Unknown single-gene or polygenic disorders, involved in fundamental cellular and reproductive processes, can cause idiopathic recurrent euploid losses. Theoretically single gene abnormalities, along with balanced chromosomal abnormalities, can be inherited by offspring and leads to RSA as a Mendelian disorder in pedigree members, hereditary RSA (13-15). 

In other hand, hereditary RSA is a genetically heterogeneous phenomenon and occurs either as autosomal dominant, autosomal recessive or X linked disorder. Up to now several single gene disorders for example hydrops fetalis result from alpha thalassemia and thrombophilic factors, have been identified to cause or increase the risk of hereditary RSA (16, 17). However, many single-gene disorders inherited in pedigrees of recurrent spontaneous abortion are remained to be identified. 

Linkage mapping study of hereditary RSA pedigrees, can lead to the identification of genetic factors responsible for such complications. For assessment of genetic linkage, linkage analysis was performed. The present study is the first one to investigate linkage to known RSA susceptibility locus in Mashhad population which is characterized by its heterogenous genetic background. Regarding the non-random X inactivation in women with repetitive miscarriage, it has been concluded that probably there are several loci on the chromosome X which can lead to occurrence of hereditary RSA (18, 19).

So far, several X linked disorders such as Rett syndrome, incontinentia pigmenti and some kinds of hemophilia type-A are identified as the causes of hereditary RSA pedigrees (16, 17). However there are a large number of X linked RSA pedigrees who are not affected by known X linked disorders. This means that there are some X linked loci which remain to be characterized as reason of X linked RSA. 

In this experiment, we have analyzed a large pedigree of hereditary RSA and, identified a region which may be involved in hereditary RSA by using linkage.

## Materials and methods

**Patients**

This experiment was a linkage analysis study. A large family with a five generation pedigree and 9 RSA patients was assessed in this experiment. Proband was a 30 year old woman having 5 consecutive spontaneous abortions without any live offspring. Various studies investigating recurrent abortion causing disorders, including anatomical abnormalities, immunological, hormonal, Thrombophilic and cytogenetic abnormalities did not show any abnormality. In addition, 8 more affected and 7 non affected women along with 11 healthy men were genotyped in this family. 

**DNA genotyping**

After completing the informed consent, 5 to 10 ml of brachial venous blood was collected in EDTA tubes. DNA was extracted from lymphocytes by a standard salting out method. 11 short tandem repeats (STRs) loci (DXS101, DXS143, DXS6789, DXS6800, DXS6807, DXS7130, DXS7132, DXS7133, DXS7423, HPRTB, GATA31E08) which scattered through the X chromosome with an average spacing of 10 cM were selected for linkage analysis. 

Microsatellites were genotyped by using of PCR amplification (primers were listed in table I). 100ng of extracted DNA was applied as template in 25μl reaction mixture containing 1x PCR buffer, 50 μM of each dNTP, 2mM MgCl_2_, 0.2 μM of each primer and 1 unit of Taq HotStart DNA polymerase. PCR reaction was performed in 2720 ABI thermal cycler which is programmed for 35 cycles of denaturation at 94^o^C for 40s, 64^o^C for 40s and 72^o^C for 2min; an additional step of denaturation at 95^o^C for 10min preceded the first cycle and another step of extension at 72^o^C for 7 min was extended at the end of reaction. PCR products were resolved using 10% Polyacrylamide Gel Electrophoresis (PAGE). According to total number of alleles in pedigrees each allele was numbered from 1 (shortest allele) to 6 (longest allele) and each patient woman receives a tow number code for each STR locus. Since men are hemi-zygote for X linked genes they receive unicharacter code. 

**Statistical analysis**

Two point parametric Linkage analysis was performed by EasyLinkage Plus v5.02 package (http://sourceforge.net/projects/easylinkage/) using Superlink v1.6 program. Pedigree was analysed as recessive model and LOD scores were obtained at 0.05 recombination increment steps until to recombination value of 0.45. Penetrance of disease and disease allele frequency were given 99% and 0.001, respectively. STR alleles were set to codominant and Map order and genetic inter-STRs distances were taken from the Marshfield STRP map.

**Table I:** PCR primer sequences in this study

Locus X-STRs	Primer sequence		Approximate PCR Product size (pb)
DXS7132	CCCTCTCATCTATCTGACTG		110-134
		GCCAAACTCTATTAGTCAAC	
DXS6800	GTTGAAATATTGGGGGCTGGT		158-186
		ATTGTGGGACCTTGTGATTGTG	
DXS6789	GTTGGTACTTAATAAACCCTCTTT		139-157
		AAGAAGTTATTTGATGTCCTATTGT	
DXS7133	GCTTCCTTAGATGGCATTCA		106-134
		CTTCCAAGAATCAGAAGTCTCC	
HPRTB	CTCTCCAGAATAGTTAGATGTAGG		238-252
		TCACCCCTGTCTATGGTCTCG	
GATA31E08	GAGCTGGTGATGATAGATGAT		132-152
		GACAGAGCACAGAGATACATG	
DXS7423	GTCTTCCTGTCATCTCCCAAC		200-228
		GACCTTGGCCTTTGTCTCC	
DXS101	TCAGTCCAAATATCTCCCTTCAA		220-232
		CAAATCACTCCATGGCACA	
DXS6807	GACCAATGATCTCATTTGCA		264-288
		AAGTAAACATGTATAGGAAAAAGCT	
DXS143	ACAGAAGTTCAGCTCTTCAG		300-316
		GGATCCTCACTAAATGAGGATGG	

## Results

**Figure 1:**
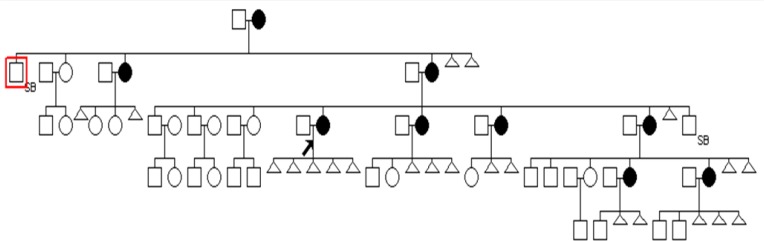
This figure shows analyzed pedigree. Proband (individual III8) was a woman with five constitutive abortions with no live birth.

**Figure 2:**
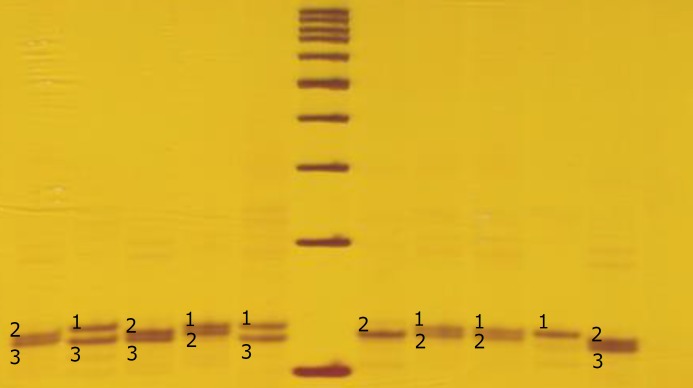
PAGE results of DXS7133 PCR products. Lengths of presented bands vary between 122-130bp. 100bp DNA size marker was used to estimation of PCR product bands.

Pedigree is shown in figure 1 with individual III8 as Proband. In this study X linked STRs genotype of pedigrees members was determined using PCR-PAGE technique. As shown in figure 2 genotype of each member was shown as PCR product bands on polyacrylamide gel. 

**Figure 3:**
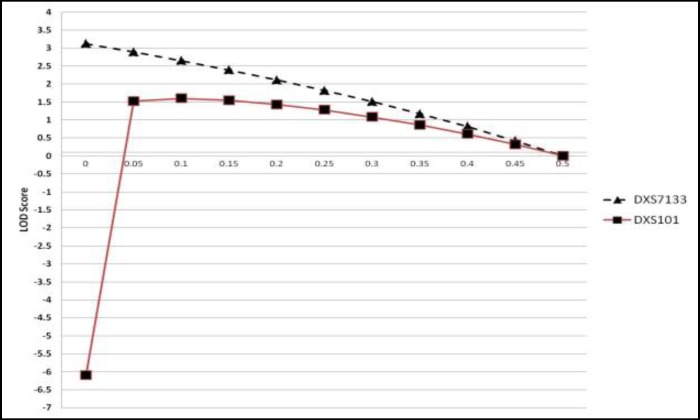
LOD scores of DXS101 and DXS7133 in different θ. LOD score higher than 1 suggests linkage presence and LOD score higher than 3 confirm linkage between STR and RSA. Results indicate that RSA causing locus is very near to DXS7133 STR locus

Genotype of pedigree members is shown in table II. Linkage analysis was performed for each locus at 0.05 recombination increment steps until to recombination value of 0.45 and results were illustrate as Z score curve. Evidence of linkage was observed to markers DXS7133 and DXS101 with maximum LOD of 3.12 and 1.60, respectively (Figure 3). There was no linkage between other STR markers and RSA. None of STR loci between pTelomer and DXS101 (DXS143, DXS6807, DXS7132, DXS6800, DXS6789) have LOD higher than 0.89. 

In other hand STR between qTelomer and DXS7133 (DXS7130, DXS7423, HPRTB, GATA31E08) does not show any evidence of linkage (Figure 5).

**Table II:** Genotype of analyzed pedigree members for each STR locus. Male members have only one allele

Family member	DXS6807		DXS7132		DXS6800		DXS6789		DXS101		DXS7133		HPRTB		GATA31E08		DXS7423		DXS143		DXS7130	
1	1	1	2	2	2	2	1	1	1	2	2	2	2	2	1	1	1	2	1	2	3	2
2	-	1	-	2	-	2	-	1	-	1	-	2	-	2	-	1	-	2	-	2	-	3
3	1	2	2	2	1	2	1	2	2	3	1	2	2	2	1	1	1	2	1	2	3	1
4	1	1	2	2	1	2	1	1	1	2	2	2	2	2	1	1	2	2	2	2	2	1
5	-	2	-	2	-	2	-	2	-	2	-	1	-	2	-	1	-	2	-	3	-	1
6	1	1	2	2	1	1	1	2	1	3	1	2	2	2	1	1	2	2	1	3	2	3
7	-	2	-	1	-	2	-	2	-	1	-	1	-	1	-	1	-	1	-	1	-	1
8	-	1	-	2	-	2	-	1	-	1	-	2	-	2	-	2	-	2	-	2	-	1
9	-	1	-	2	-	2	-	1	-	2	-	2	-	2	-	1	-	2	-	3	-	3
10	-	1	-	2	-	2	-	1	-	2	-	2	-	2	-	1	-	1	-	3	-	3
11	1	2	1	2	1	2	1	2	1	2	1	2	1	2	1	1	1	1	1	3	2	3
12	1	1	1	2	1	2	1	2	1	2	1	2	1	2	1	2	1	1	1	3	2	3
13	-	2	-	2	-	2	-	2	-	2	-	2	-	2	-	2	-	1	-	1	-	3
14	1	1	2	2	2	2	1	2	1	2	2	2	2	2	1	1	2	2	3	1	2	3
15	1	1	1	2	1	2	1	2	1	2	1	2	1	2	1	1	2	2	1	2	1	3
16	-	1	-	2	-	2	-	2	-	2	-	2	-	2	-	1	-	2	-	1	-	1
17	2	2	2	2	1	2	1	2	2	2	2	2	2	2	1	1	1	1	3	1	2	3
18	2	1	2	2	2	2	1	1	2	3	2	2	2	2	1	2	1	1	2	1	2	2
19	1	1	2	2	1	2	1	2	1	2	1	2	2	2	1	2	1	1	2	1	2	2
20	-	1	-	2	-	1	-	4	-	1	-	2	-	2	-	4	-	1	-	2	-	1
21	1	1	2	3	1	1	1	3	1	1	2	3	2	2	2	4	1	1	1	1	1	1
22	1	1	2	2	1	1	3	4	1	1	2	3	2	2	2	4	1	1	2	1	2	1
23	1	1	3	2	1	1	1	4	1	1	2	2	2	2	4	4	1	1	4	3	2	3
24	1	1	2	2	1	1	3	4	1	1	2	2	2	2	2	4	1	1	1	3	1	3
25	-	1	2	2	-	2	-	1	-	1	-	1	-	2	-	3	-	1	-	3	-	1
26	1	1	1	2	1	2	1	3	1	1	1	3	1	1	2	3	1	1	1	3	1	3
27	-	1	-	2	-	2	-	3	-	1	-	3	-	2	-	2	-	1	-	1	-	1

**Figure 4:**
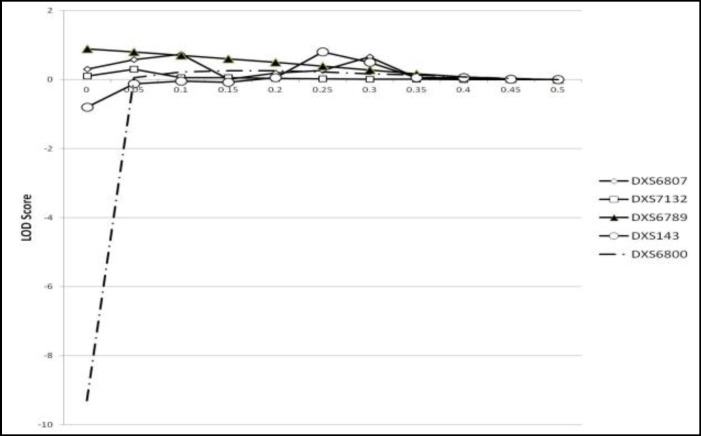
LOD scores STR loci between DXS101 and pTelomer. There is no evidence suggesting linkage presence

**Figure 5:**
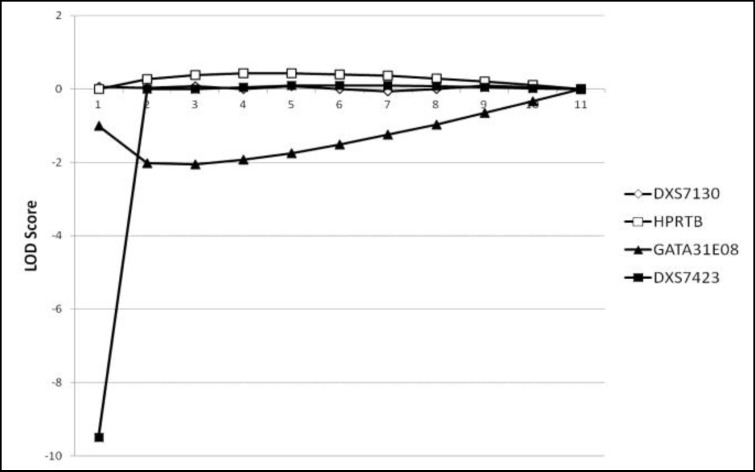
LOD scores STR loci between DXS7133 and qTelomer. There is no evidence suggesting linkage presence

## Discussion

Recurrent pregnancy loss can be caused by several factors involved in fundamental events during human reproduction. Anatomical, immunological, hormonal and infectious factors along with known genetic factors are involved in 50% of cases, however remaining 50% of abortions has not any known reason and named as idiopathic abortion. In recent decade increasing number of evidence indicate that genetic disorders may be responsible in major part of idiopathic abortions. 

Although RSA often occur as isolated case, however, sometimes hereditary RSA found with apparent Mendelian inheritance mode. These RSA pedigrees have important role in identification of new RSA etiologic loci. Linkage analysis can be performed on these pedigrees to candidate loci as genetic factors of these RSA cases. Moreover, in some experiments on X linked RSA pedigrees some loci such as Incontinentia pigmenti, Rett syndrome and hemophilia were identified to be linked with RSA (16, 17). However a large number of hereditary RSA pedigrees are remained to be analyzed.

Between RSA pedigrees, X linked RSA pedigrees has interesting characteristics which encourage linkage analysis. As possible locus located on X chromosome, all of analyzed polymorphic markers can be confined on this chromosome. As a result, the number of needed markers decrease in compare with autosomal pedigrees and performing linkage become very easy. Furthermore, it has been suggested that a significant portion of isolated RSA cases may have X chromosome disorders. These disorders lead to reduction of fitness in cell which has defected X chromosome as active one. This leads to deviation of 50:50 ratio of cells inactivating either X chromosome, a phenomena known as Skewed X inactivation.

Several studies suggest that Skewed X inactivation, defined as 90% inactivation of one parental allele, has association with increasing of recurrent abortion (10, 18, 19). In recent decades, several studies investigate the presence of Skewed X chromosome inactivation in women with RSA. Bagislar and colleagues demonstrate that Skewed X inactivation has a significant association with RSA in an X linked RSA pedigree (10, 18). Results of these studies show relatively high inconsistency, which may be a result of different etiologies of RSA in analyzed women.

In this work, we present the genotyping results of 27 members (11 males and 16 females) of an X linked RSA pedigree from a village located near Mashhad, Iran. Loci DXS7132, DXS6800, DXS6789, DXS7133, HPRTB, GATA31E08, DXS7423, DXS101, DXS6807, DXS143, and DXS7130 have been investigated (20, 21). All analyzed markers are STRs spread over an extensive region covering almost all the X chromosome, with the exception of the pseudoautosomal region. All markers investigated are distant from each other by more than 4 Mb (http://research.-marshfieldclinic.org/ genetics/ Map_Markers/ maps/IndexMap-Frames.html). 

In this study we report mapping of a novel region on X chromosome (Xq23) in relation to X linked recurrent abortion following linkage analysis. Our result is in agreement with previous reports of linkage for autism loci; particularly those reported by Kilpinen *et al* and linkage for X-linked dominant genodermatosis incontinentia pigmenti (22, 23). This region is tracked by markers DXS101 and DXS7133 at approximately 10 cM distance. Our results suggest a possible gene on chromosome Xq23 that might be responsible for X-linked. 
